# Extraction, Characterization, and Evaluation of the Cytotoxic Activity of Piperine in Its Isolated form and in Combination with Chemotherapeutics against Gastric Cancer

**DOI:** 10.3390/molecules28145587

**Published:** 2023-07-22

**Authors:** Ingryd Nayara de Farias Ramos, Monique Feitosa da Silva, Jefferson Marcio Sanches Lopes, Jordy Neves Cruz, Fabrine Silva Alves, José de Arimatéia Rodrigues do Rego, Marcondes Lima da Costa, Paulo Pimentel de Assumpção, Davi do Socorro Barros Brasil, André Salim Khayat

**Affiliations:** 1 Oncology Research Center, Federal University of Pará, Belém 66075-110, PA, Brazil; monique.silva@icb.ufpa.br (M.F.d.S.); assumpcaopp@gmail.com (P.P.d.A.); khayatas@gmail.com (A.S.K.); 2 Department of Physics, Federal University of Roraima, Boa Vista 69310-000, RR, Brazil; lopesjefferson01@yahoo.com.br; 3 Institute of Technology, Federal University of Pará, Belém 66075-110, PA, Brazil; jorddynevescruz@gmail.com (J.N.C.); davibb@ufpa.br (D.d.S.B.B.); 4 Graduate Program in Pharmaceutical Innovation, Federal University of Pará, Belém 66075-110, PA, Brazil; fafa.bine@yahoo.com.br; 5 Graduate Program in Science and Environment, Federal University of Pará, Belém 66075-110, PA, Brazil; jrego@ufpa.br; 6 Institute of Geosciences, Federal University of Pará, Belém 66075-110, PA, Brazil; mlc@ufpa.br; 7 Institute of Biological Science, Federal University of Pará, Belém 66075-110, PA, Brazil

**Keywords:** piperine, extraction and purification, green extraction, cytotoxicity, gastric cancer

## Abstract

Gastric cancer is one of the most frequent types of neoplasms worldwide, usually presenting as aggressive and difficult-to-manage tumors. The search for new structures with anticancer potential encompasses a vast research field in which natural products arise as promising alternatives. In this scenario, piperine, an alkaloid of the *Piper* species, has received attention due to its biological activity, including anticancer attributes. The present work proposes three heating-independent, reliable, low-cost, and selective methods for obtaining piperine from *Piper nigrum* L. (Black pepper). Electronic (SEM) and optical microscopies, X-ray diffraction, nuclear magnetic resonance spectroscopies (^13^C and ^1^H NMR), and optical spectroscopies (UV–Vis, photoluminescence, and FTIR) confirm the obtention of piperine crystals. The MTT assay reveals that the piperine samples exhibit good cytotoxic activity against primary and metastasis models of gastric cancer cell lines from the Brazilian Amazon. The samples showed selective cytotoxicity on the evaluated models, revealing higher effectiveness in cells bearing a higher degree of aggressiveness. Moreover, the investigated piperine crystals demonstrated the ability to act as a good cytotoxicity enhancer when combined with traditional chemotherapeutics (5-FU and GEM), allowing the drugs to achieve the same cytotoxic effect in cells employing lower concentrations. These results establish piperine as a promising molecule for therapy investigations in aggressive gastric cancer, both in its isolated form or as a bioenhancer.

## 1. Introduction

Gastric cancer occupies the fifth position among the most common types of cancer worldwide [1,2]. Generally diagnosed late at advanced stages of the disease, this type of tumor inherits high heterogeneity, which is a determinant feature for its phenotypic aggressiveness [3] and high mortality rates. The limitations faced in current therapy are additional concerns since they can lead to severe side effects and resistance to multiple drugs, implying high recurrence rates and relative therapeutic failure [4,5]. This set of factors reveals that gastric cancer is still a clinically challenging disease, with low effective treatment options, especially concerning aggressive tumors [6]. Therefore, searching for more effective treatments based on new molecules is necessary for improving therapy.

Natural products are promising sources of pharmacologically active molecules capable of interacting with cell membrane receptors, nucleic acids, and other mechanistic pathways [7,8,9]. Their potential biological activity has been intensively investigated in preclinical and clinical studies [10], revealing promising effects related to a wide range of plant-derived compounds and secondary metabolites such as terpenes, phenolic compounds, and alkaloids [11]. Indeed, natural molecules such as taxol, artemisinin, and vinblastine [12] are currently employed in a variety of drugs for the clinical treatment of different diseases, being plant-derived species of particular importance [10].

**Figure 1:**
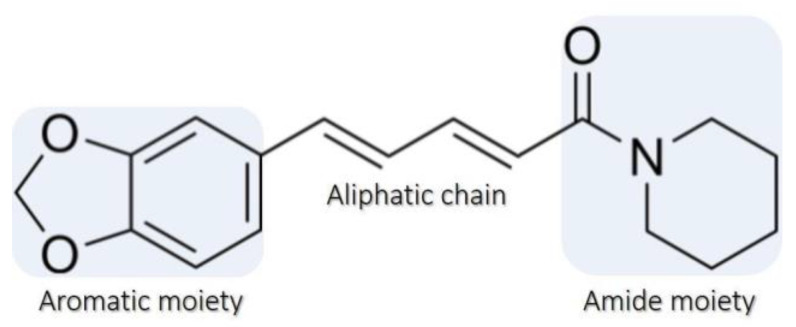
Chemical Structure of the (2E,4E)-5-(1,3-benzodioxol-5-yl)-1-(piperidin-1-yl) penta-2,4-dien-1-one molecule (piperine). The molecule is composed of the linking between the amide and aromatic moieties via an aliphatic chain.

Among the vast number of natural structures, piperine (Figure 1), a remarkable active alkaloid generally found in species of the *Piper* family, such as *Piper longum* (long pepper) and *Piper nigrum* L. (black pepper), and responsible for their pungencies, has been studied recently [13,14]. The relative simplicity of its obtention makes piperine commercially attractive. In this regard, controlling physicochemical parameters allows the establishment of different extraction approaches, such as the soxhlet method, which involves sample heating and requires a low amount of organic solvents [15], being a relatively inexpensive way for obtaining piperine. However, compared to other approaches, the soxhlet method demands a long extraction time [16]. Methods based on external agents such as ultrasound [17], microwaves [18,19], maceration [20,21], and others [22] are also reported in the literature. In all cases, piperine isolation relies on a recrystallization step, resulting in yellow-colored needle-shaped microscopic crystals obtained with good yields and high purity [16,23].

Piperine exhibits several biochemical and pharmaceutical properties, being capable of interacting with chemically and functionally diverse molecular targets [24], as confirmed by in vitro and in vivo studies. The attention attributed to the study of this molecule is mainly due to its biological properties, such as anti-inflammatory [25], antimicrobial [26,27], antidepressant [28,29], antifungal [30,31], and anticancer properties [32,33]. In addition, piperine is regarded as a target compound, as it is the principal molecule in black pepper extract and belongs to the class of alkaloids, which have already proven anticancer activity through chemical interactions and activation of different pathways in tumor cells [34]. Specifically, regarding the anticancer activity of piperine, recent observations show that its mechanism of action is multiple and involves the activation of cellular and molecular signaling pathways, with programmed cell death, decreased migration and invasion, and reduced cell proliferation [35]. Notably, in addition to acting in isolation, piperine can increase the bioavailability of other compounds [36,37], favoring its use as a food supplement [38] and in combination with drugs from different classes (including current chemotherapy agents) [39,40].

This set of factors places piperine at the heart of the search for new therapeutic approaches for gastric cancer. In the present work, we obtained piperine via three heating-independent methods and characterized the samples employing several techniques. Moreover, the molecule’s in vitro cytotoxic activity in its isolated form and combined with the chemotherapeutics 5-Fluorouracil (5-FU) and Gemcitabine (GEM) are evaluated. The cytotoxicity experiments were counted with five Brazilian Amazon native gastric cancer lineages (AGP01, AGP01 PIWIL1 KO, ACP02, and ACP03), with the models bearing a variety of molecular profiles, including metastatic biomarkers and genetic modifications [41]. We present novel discussions on the in vitro cytotoxicity of piperine against different gastric cancer lineages, demonstrating the relationship between the molecule’s cytotoxic activity and the phenotypes of the evaluated models. The results show that piperine has pronounced activity against cells with a higher level of aggressiveness. In addition, we also showed that besides acting in isolation in cancer models, piperine enhances the activity of commercial chemotherapy drugs.

## 2. Results and Discussion

### 2.1. Isolation and Characterization of Piperine

#### 2.1.1. Microscopy and X-ray Diffraction Analysis

**Figure 2:**
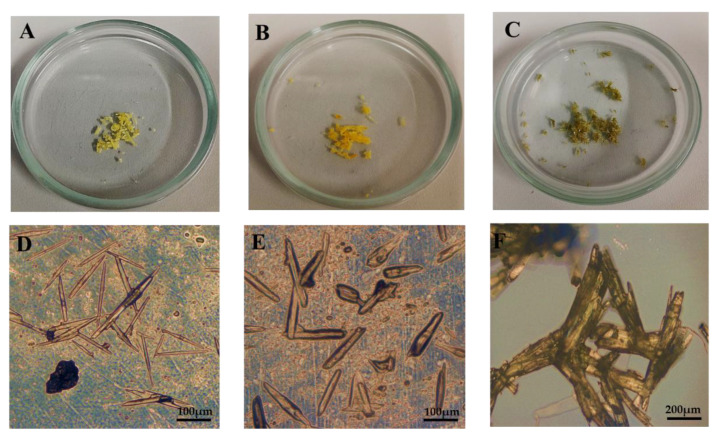
Photographs (**top**) and micrographs obtained by optical microscopy (**bottom**) of the P1 (**A**,**D**), P2 (**B**,**E**), and P3 (**C**,**F**) samples.

The recrystallization of piperine from *Piper nigrum* L. extracts employing the three heating-independent approaches proposed in this work revealed reliable and low-cost methodologies. The obtained solid-state yellow-colored samples (P1, P2, and P3), shown in Figure 2, exhibited good structural and color stability at room temperature for at least 120 days of storage.

Optical microscopy analysis of these samples (Figure 2D–F) revealed that they are composed of needle-shaped crystals, which along with their characteristic yellow color are compatible with the reported piperine crystal [26,42,43]. Comparing the micrograph of P3 with the results from P1 and P2, we observe that although aggregated, this sample is still composed of needle-shaped crystals. This result confirms that adopting only pure water as a recrystallization mediator is still effective for obtaining the piperine crystals, even without employing KOH in the process.

**Figure 3:**
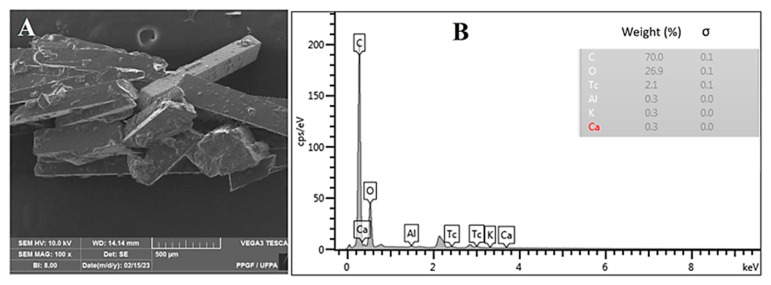
(**A**) SEM micrograph and (**B**) EDS spectrum of P2.

As discussed later, further characterization with other techniques (e.g., XRD, NMR, and FTIR) reveals no significant differences between the P1, P2, and P3 samples. This trend led us to employ P2 as a representative for the other samples in the scanning electron microscopy (SEM) and energy dispersive spectroscopy (EDS) analysis. In agreement with the literature [26,44,45], in Figure 3A, it is demonstrated that the crystals in P2 follow the monoclinic system, with longitudinal lengths varying between ~100 μm and ~800 μm. The EDS analysis (Figure 3B) reveals that the sample is mainly composed of carbon (70.0% of relative weight) and oxygen (26.9% of relative weight) present in abundance in piperine which is supportive of its level of purity.

**Figure 4:**
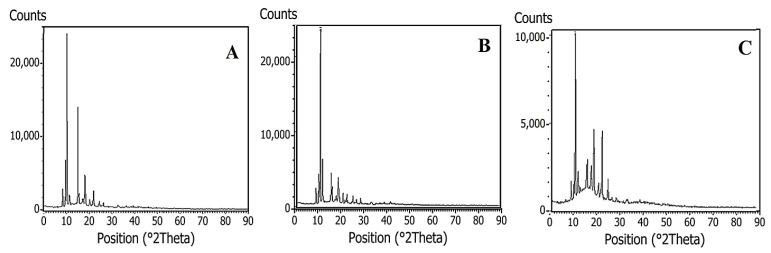
XRD diffractograms of (**A**) P1, (**B**) P2, and (**C**) P3 piperine samples.

The XRD analysis reveals several atomic planes in all samples, with peaks identified between 10° and 50° (Figure 4). The observed diffractograms agree with the literature supporting the assignment of a monoclinic regime presented in piperine crystals [26,44,45]. As shown in Figure 4C, the XRD diffractogram of sample P3 exhibits an increased baseline, which is suggestive of sample amorphousness and can correlate with the crystal aggregation observed by optical microscopy.

#### 2.1.2. Spectroscopy Analysis

**Figure 5:**
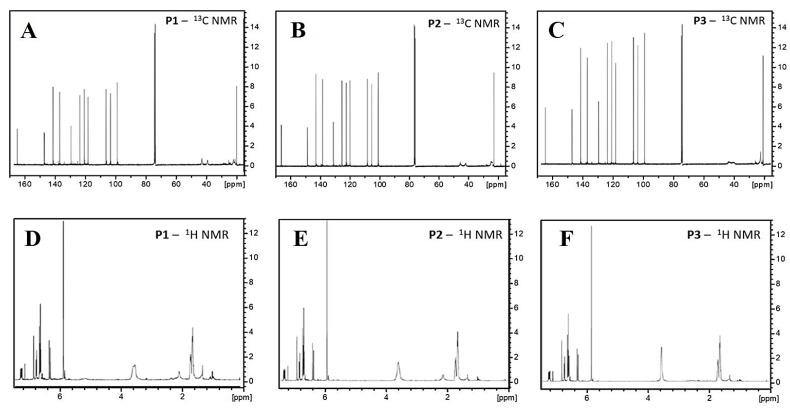
^13^C NMR (**top**) and ^1^H NMR (**bottom**) spectra of P1 (**A**,**D**), P2 (**B**,**E**), and P3 (**C**,**F**) piperine samples.

Employing NMR spectroscopies (^13^C and ^1^H), we could track signatures from piperine’s aromatic, amide, and aliphatic groups in all samples (P1, P2, and P3). Firstly, considering the ^13^C NMR spectra, depicted in Figure 5A–C, we verify that the π carbons of the aromatic portion of piperine can be assigned [42,46,47] to the transitions located at ca. 120 ppm, 123 ppm, and 144 ppm, while the O-C-O and C=C-O bonds correlate with the transition at ~125 ppm and the transitions at ~28 ppm, ~45 ppm, and ~48 ppm, respectively. The amide portion of piperine [42,46,48] is associated with the transitions at ~100 ppm (N-C=O), ~109 ppm, and ~150 ppm. Finally, the transitions located at ~132 ppm, ~139 ppm, and 166 ppm correlate with the aliphatic chain [42,47,49]. The transition at ~78 ppm is assigned to the reference solvent (CDCl_3_).

The same set of functional groups is observed in the ^1^H NMR spectra, as shown in Figure 5D–F. The aromatic moiety is related to the transitions located at ca. 6.6 ppm, 7.3 ppm, 1.68 ppm (C=C-O), 1.73 ppm (C=C-O), and 3.6 ppm (C=C-O) [42,46,47]. The transition at ~5.9 ppm is characteristic of the N-C=O bond, which addresses the presence of the piperine’s amide moiety. The signatures at ~6.8 ppm and ~7.1 ppm evidence the aliphatic chain. The signal associated with the reference solvent (CDCl_3_) is located at ~7.5 ppm. According to all the presented results, there are no significant differences between the NMR spectra of the P1, P2, and P3 samples. Furthermore, no additional signals, other than the transitions specific from piperine and the adopted solvent, were observed, suggesting that if impurities are present in the crystals, they display relatively small concentrations regarding the concentration of piperine.

**Figure 6:**
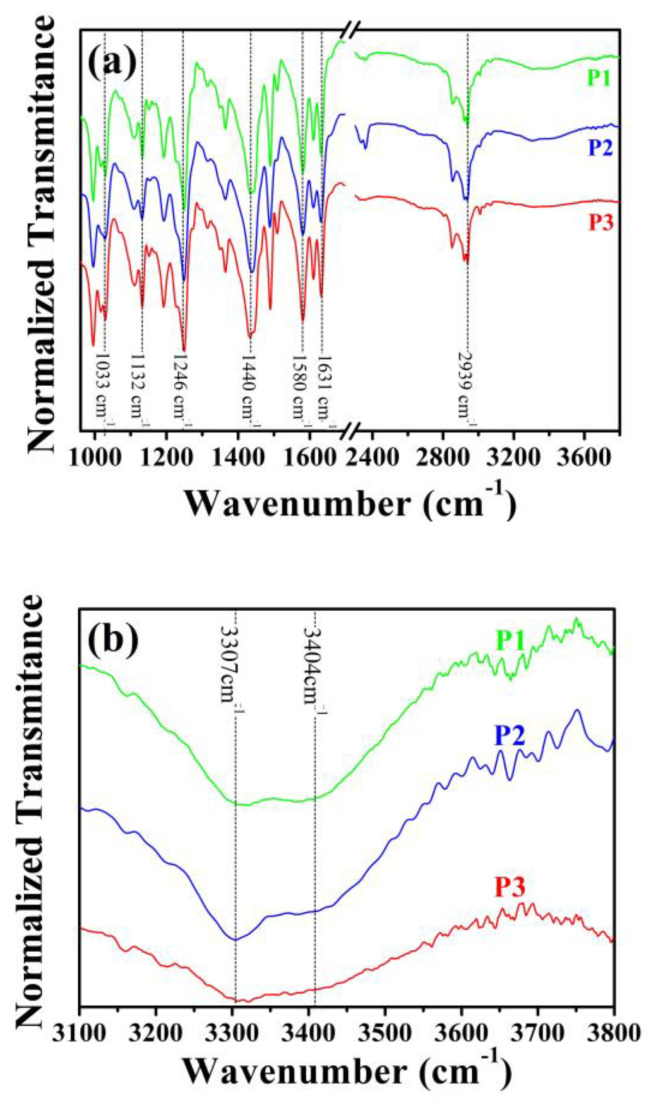
Fourier Transform Infrared spectra of P1 (green solid line), P2 (blue solid line), and P3 (red solid line) piperine samples (**a**) in the range 950–3800 cm^−1^. In (**b**), the region of 3100–3800 cm^−1^ is emphasized to track possible remnant contents of water and KOH in the solid samples.

The vibrational spectra of P1, P2, and P3 share significant similarities (Figure 6), with all samples bearing vibrational modes located at 1033 cm^−1^ (symmetric =C-O-C stretching), 1132 cm^−1^ (asymmetric =C-O-C stretching), and 1580 cm^−1^ (aromatic stretching of the benzene ring) [18,47,50] which identify the aromatic portion of the piperine structure. The amide portion is associated with the vibrational mode at 1631 cm^−1^ (C=O-N bond) [46,51]. Finally, the high-energy vibrational mode centered at 2940 cm^−1^ [42,46,47] assigns the C-H stretching in the aliphatic chain.

Since we measured the samples in their pristine form (solid-state), FTIR spectroscopy also allows tracking further information on the presence of possible remnant undesired substances. Of particular concern, the recrystallization of piperine could trap remnant reagents such as water and KOH inside or outside the solid samples. As reported in the literature, the water molecule exhibits active vibrational modes at ~3300 cm^−1^, correlated with the symmetric and asymmetric stretching of its O-H bond [52]. Regarding KOH, Snyder and co-workers [53] demonstrated that at room temperature (~23 °C), a broadband vibrational mode at ~3600 cm^−1^ arises from its O-H stretching. After conducting a careful evaluation in the high energy region of our spectra, we verified the existence of low-intensity signals at ~3307 cm^−1^ and 3404 cm^−1^ (Figure 6b) assigned to the vibrational modes of water [52]. Notably, the low intensity of these peaks regarding the piperine’s vibrational modes suggest reduced amounts of moisture in all samples. No evidence of KOH signatures is verified, endorsing the absence of remnant reagents in the samples.

**Figure 7:**
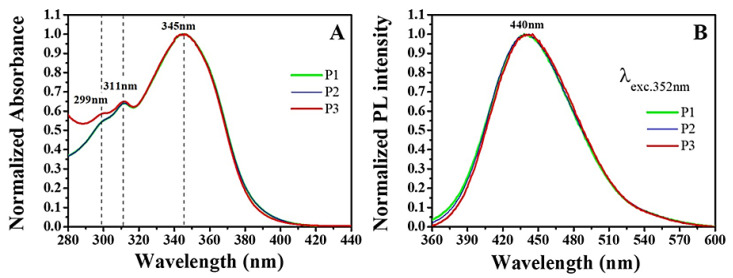
(**A**) UV–Vis absorption and (**B**) steady-state PL (λexc. 352 nm) spectra of P1 (green solid line), P2 (blue solid line), and P3 (red solid line) piperine samples dissolved in P.A. dimethylsulfoxide.

From UV–Vis absorption spectroscopy, we verify that P1, P2, and P3 display characteristic absorption bands centered at 299 nm, 311 nm, and 345 nm, respectively (Figure 7A). These features are consistent with previously reported data [54,55,56] and can be associated with electronic and vibronic transitions in piperine. The steady-state photoluminescence (PL) spectra of the samples display a characteristic emission band centered at 440 nm, which reportedly arises from piperine [57] (Figure 7B). Notably, no other emission bands are observed in Figure 7B, suggesting that piperine is the only emissive compound in P1, P2, and P3 samples. Comparing the most intense absorption and PL bands, we verify Stokes shifts of ~6260 cm^−1^ in all samples, which is in agreement with the literature [57].

### 2.2. Piperine Cytotoxicity in Gastric Cancer Models

#### 2.2.1. Isolated Piperine

**Figure 8:**
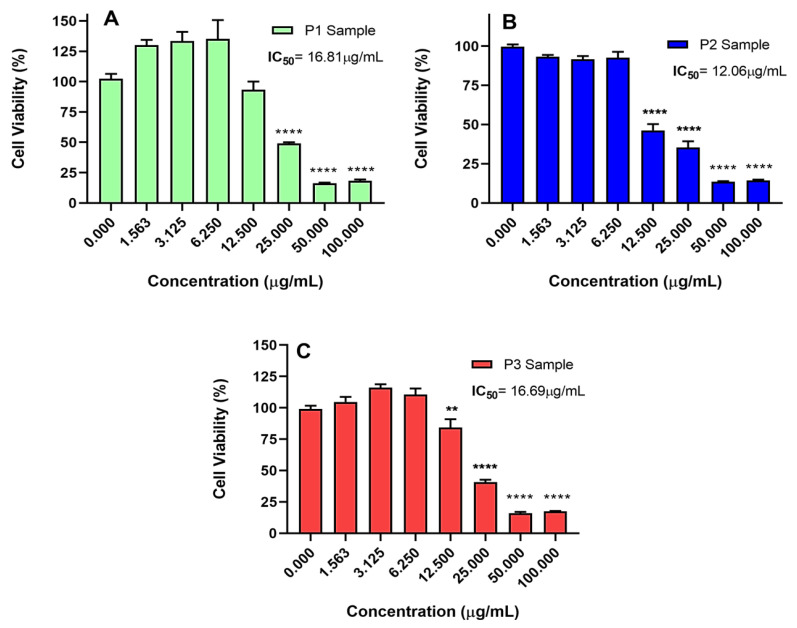
Cell viability of AGP01 cell line after 72 h of treatment with (**A**) P1, (**B**) P2, and (**C**) P3. The results are expressed in percentages regarding the untreated control. Each point is equivalent to the mean ± standard deviation of three replicates. Statistical analysis was performed with ANOVA followed by Bonferroni’s posttest. Significant differences: ** *p* < 0.005, **** *p* < 0.0001.

The cytotoxicity of the P1, P2, and P3 samples was evaluated for the AGP01 lineage, which originates from peritoneal metastasis and has genetic alterations capable of providing more resistance to the action of substances or chemotherapy [58,59,60]. As observed by the MTT assay, all piperine samples are cytotoxic against the tested model, with IC50 values of 16.81 µg/mL (P1), 12.06 µg/mL (P2), and 16.69 µg/mL (P3), as shown in Figure 8. Furthermore, P1, P2, and P3 caused a decrease in cell viability as a function of the increase in concentration, demonstrating a concentration dependency. The results also show that P2 displays higher cytotoxic activity, with a lower IC50 value and with a statistically significant reduction in cell viability, starting from a concentration of 12.5 µg/mL (Figure 8B). Other studies have also demonstrated that piperine causes a concentration-dependent reduction in cell viability of rectal [61], cervical [62], and prostate cancer cells [63]. However, the IC50 of piperine in these studies lies around 30 µg/mL, showing that piperine is even more active in metastatic gastric cancer.

**Figure 9:**
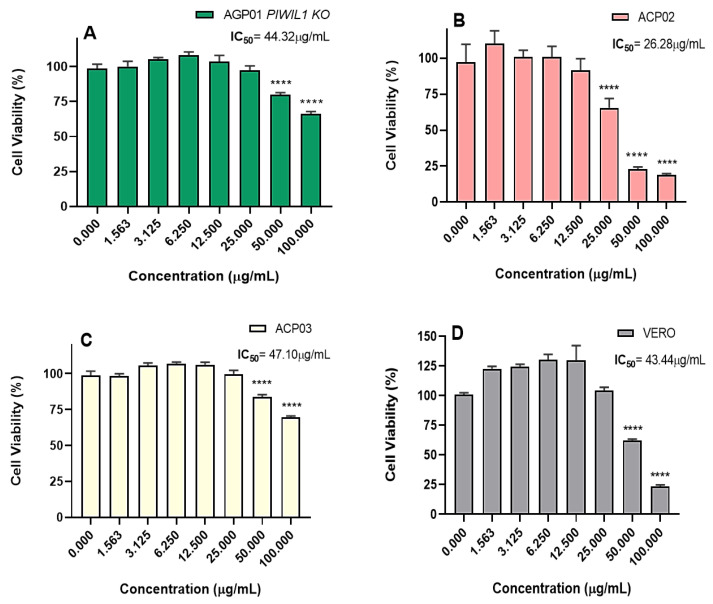
Cell viability of different gastric cancer lineages: (**A**) AGP01 *PIWIL1 KO*, (**B**) ACP02, (**C**) ACP03, and (**D**) a non-tumor cell (VERO), after 72 h of treatment with P2. The results are expressed in percentages concerning the untreated control. Each point is equivalent to the mean ± standard deviation of three replicates. Statistical analysis was performed with ANOVA followed by Bonferroni’s posttest. Significant differences: **** *p* < 0.0001.

From the initial screening, the P2 sample (which showed the best activity among all piperine samples) was evaluated for cytotoxicity in three other gastric cancer lineages: a diffuse model (ACP02), an intestinal model (ACP03), and a metastasis model with an inactivated *PIWIL1* gene (AGP01 *PIWIL1 KO*). Additionally, aiming to investigate the selectivity of the compound, a non-tumor cell line (VERO) was also evaluated. The results show that P2 is cytotoxic in all tested models. However, there are very significant differences in the obtained IC50 values. These values were 44.32 µg/mL, 26.28 µg/mL, 47.10 µg/mL, and 43.44 µg/mL for AGP01 *PIWIL1 KO*, ACP02, ACP03, and VERO lineages, respectively (Figure 9). The level of cell viability was also heterogeneous between the cell models, with most of them showing significant differences regarding the negative control only in the two highest tested concentrations (50 and 100 µg/mL) (Figure 9A,C,D). Especially for ACP02, this difference starts from 25 µg/mL (Figure 9B).

According to the results, AGP01 (Figure 8B) is the most sensitive cell to treatment with P2, presenting a lower IC50 when compared to the other tested models. Furthermore, this lineage is the only selective, regarding the non-tumor model (VERO), with a selectivity index (SI) [64,65] of 3.6, demonstrating that P2 has specific mechanisms in the tumor cells. The selective action of piperine has been reported for other cancer models, with higher cytotoxicity against tumor cells compared to normal ones [33,66]. These results show that piperine is safe for therapeutic use, being even advantageous over other non-selective tested molecules and substances of clinical use [67,68].

Multiple factors can explain this behavior, such as the intrinsic pathways of different lineages under the action of piperine. Data from the literature have already demonstrated that piperine displays distinct mechanistic actions, mainly attributed to its ability to interact with different molecular targets, including kinases [32], transcription factors [69], cell cycle proteins [70], receptors, and molecules of signaling [71], thus supporting its potential as an anticancer agent. Among the studied effects of piperine, both in vitro and in vivo, are included the induction of apoptosis [72,73], the inhibition of cell proliferation [63,74] with cell cycle arrest [70,73], and the modulation of the expression of genes and proteins involved in the processes of cell migration and invasion [75,76]. All these effects influence the outcome of carcinogenesis of various types of tumors, such as breast [77,78], cervical [62,79], and colorectal [80,81].

Considering that the IC50 of P2 on the AGP01 lineage (12.06 µg/mL) was almost four times lower than on the AGP01 *PIWIL1 KO* lineage (44.32 µg/mL), we can attribute such better cytotoxicity to the inactivation of the *PIWIL1* gene, since that is the only difference between the lineages. According to the literature, the inactivation of the *PIWIL1* gene is associated with a decrease in the cell’s ability to migrate and invade, promoting a change in the gene expression related to these processes [82,83]. Therefore, considering the higher cytotoxicity of P2 in AGP01, which has all these genes activated, and the data reported on the action of piperine in other types of tumors [33,84,85], it is possible to suggest that the effects of piperine in gastric cancer may be closely related to the presence of specific pathways of cell migration and invasion. Moreover, piperine seems more effective for treating tumor cells with high levels of aggressiveness.

Regarding the action of P2 in primary gastric cancer models (ACP02 and ACP03), the cytotoxicity was higher in the diffuse type model (ACP02; IC50: 26 µg/mL) concerning the intestinal type (ACP03; IC50: 47 µg/mL). Diffuse gastric cancer (CGD) exhibits a more aggressive characteristic, with undifferentiated cells, mutations, and changes in the expression of genes involved in the epithelial-mesenchymal transition (EMT) [86,87,88]. In this scenario, we can infer that the more pronounced cytotoxicity of P2 on the ACP02 lineage once again shows that piperine has a better action on cancer cells with a more aggressive phenotype and that possibly its mechanism of action is involved with the migration and invasion pathways, supporting its potential use. Corroborating our results, Gunasekaran and co-workers showed that piperine has a remarkable in vitro action on hepatocellular carcinoma, an aggressive and difficult-to-treat tumor [89]. In breast cancer with a triple-negative phenotype, a similar result has been reported, with piperine showing antiproliferative activity [90]. This tumor is considered one of the most aggressive breast cancers due to its rapid growth and increased probability of generating metastasis [91], which endorses our hypothesis that piperine is more active in more aggressive cells capable of expressing migration and invasion pathways.

Following the results, new opportunities for evaluating therapeutic interventions in gastric neoplasms with aggressive phenotype arise, which is appropriate since they are diagnosed later, with a worse prognosis and with high mortality rates [92,93]. However, further investigations regarding the intracellular and molecular mechanisms by which piperine acts in gastric cancer are necessary for complete elucidation.

#### 2.2.2. Piperine in Association with 5-Fluorouracil and Gemcitabine

The fact that piperine has shown increased activity in cells with a more aggressive phenotype, i.e., characterized by high levels of mutations, genomic instability, and the improved ability to grow and spread [94,95], is associated with its already reported capacity to enhance the activity of other drugs opens promising opportunities. Therefore, we evaluated the cytotoxic activity against the metastatic lineage AGP01 by combining piperine with two chemotherapeutics of clinical use in gastric cancer, 5-Fluorouracil (5-FU) and gemcitabine (GEM).

**Figure 10:**
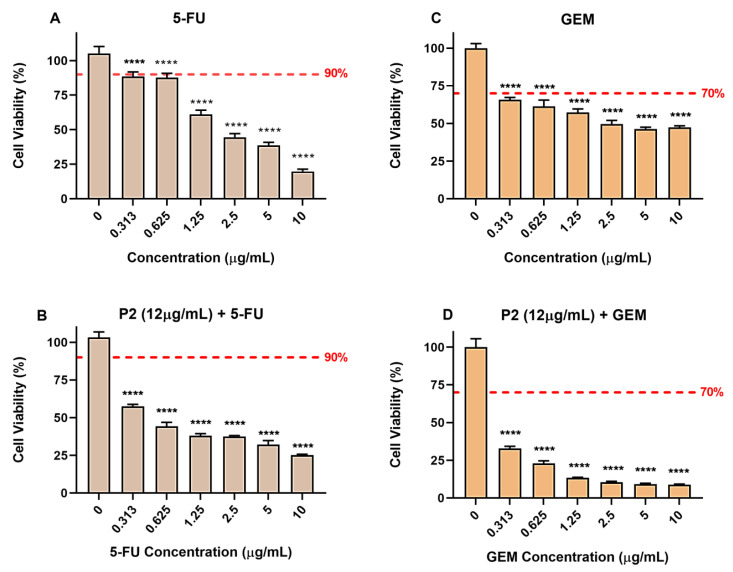
Cell viability of AGP01 cell line, after 72 h of treatment with (**A**) 5-FU, (**B**) 5-FU in combination with piperine, (**C**) GEM, and (**D**) GEM in combination with piperine. The results are expressed in percentages concerning the untreated control. Each point is equivalent to the mean ± standard deviation of three replicates. Statistical analysis was performed with ANOVA followed by Bonferroni’s posttest. Significant differences: **** *p* < 0.0001.

Regarding 5-FU, the results show that this chemotherapeutic displays concentration-dependent cytotoxicity in the tested model, leading to a reduction in its cell viability, with a significant difference in comparison to the negative control, starting at the lowest concentration tested (0.313 µg/mL). As present in Figure 10A, the viability percentage at this concentration is approximately 90%, reaching 50% only at the concentration of 2.5 µg/mL. This result shows that at lower 5-FU concentrations there is only a small variation in cell viability, with great variations requiring the highest tested concentrations.

Combining 5-FU prepared in the same concentration range (0–10 µg/mL) with a single concentration of piperine (P2), set to its IC50 value (~12 µg/mL), we observed a significant improvement in the concentration-dependent cytotoxic effect of 5-FU. As depicted in Figure 10B, the combination improves the activity of 5-FU, allowing the lowest concentration of the chemotherapeutic (0.313 µg/mL) to decrease cell viability to 50% in the presence of piperine. Since in the absence of piperine, the viability only reaches 50% for 2.5 µg/mL, this trend demonstrates that the combination effectively enhances the action of 5-FU, allowing the same cytotoxic effect in cells even employing concentrations approximately eight times lower.

The cytotoxicity evaluation of GEM followed a similar behavior. As shown in Figure 10C, GEM is cytotoxic to the AGP01 lineage over the entire concentration range tested (0–10 µg/mL). For the lowest concentration tested (0.313 µg/mL), cell viability reaches 70%. As the concentration increases, a saturation pattern is achieved (Figure 10C). However, the combination of piperine with GEM significantly enhances the cytotoxic effect. As depicted in Figure 10D, the viability of the lower concentration of GEM (0.313 µg/mL) decreases to approximately 30% in the presence of piperine. Although viability still saturates with the increase of concentration, we verify that in the combination test, the limit value is lower (~12%) concerning the viability found in the absence of piperine (~50%).

The combination with P2 increases the cytotoxic activity of GEM so significantly that the effect on gastric cancer cells at the lowest concentration of the combined curve (0.313 µg/mL) is even greater than that of the highest concentration (10 µg/mL) in the absence of piperine, where the cell viability values are 32.75% and 47.3%, respectively. This evidence shows that piperine can improve the action of GEM in gastric cancer cells considerably and that the combined treatment could decrease the employed concentration of the chemotherapeutic, causing better effects than in its isolated form.

One of the main disadvantages of current chemotherapy for gastric cancer is the increased toxicity of drugs, especially at high concentrations [96,97]. Therefore, evaluating new substances able to increase their effects at lower doses would eventually reduce this toxicity [98,99,100]. The combination of drugs in cancer therapy is a valuable treatment modality, as it increases the effectiveness of drugs, seeking to act on the main pathways in a synergistic or additive way [101]. This approach can decrease resistance to chemotherapy and provide anticancer benefits such as reduced cell proliferation and metastasis [102]. Thus, the results observed for the combination between piperine, and the chemotherapy drugs 5-FU and GEM corroborate the data already reported in the literature, which show significant effects of piperine in increasing the bioavailability of other compounds, such as antitubercular compounds [103], anti-inflammatory compounds [104], antibiotics, and even chemotherapeutics [105]. These reports demonstrate that different systems combined with piperine can have a higher impact on the bioavailability of co-administered drugs by diverse mechanisms, including inhibiting efflux transport, inhibiting intestinal and hepatic metabolism, and modulation of activity and expression of metabolic response enzymes [106].

The action of piperine combined with chemotherapeutic agents for clinical use in other types of cancer has also been reported. In breast cancer cells, piperine was combined with cisplatin and led the cells to apoptosis more effectively than the drug administered alone, reducing the toxic dose used in chemotherapy [40]. The combination of piperine and docetaxel brought an improved antiproliferative response against taxane-resistant prostate cancer in vitro and in vivo models [107,108]. Furthermore, in an in vitro model of cervical cancer (HeLa linage), piperine combination with paclitaxel demonstrated a synergistic effect, sensitizing tumor cells toward the drug’s action [109]. Therefore, chemotherapeutic systems combined with piperine are effective in the cytotoxicity of tumor cells, generating increased absorption and therapeutic efficacy of the drugs and allowing lower concentrations to produce the same or even improved effects [71,110,111]. This trend endorses our findings in gastric cancer models since piperine enhanced the activity of the tested chemotherapeutic drugs, decreasing their required effective concentrations in vitro. Because piperine exhibits multiple mechanisms in gastric cancer cells, its enhancing ability may still bring other benefits in the search for more effective treatments.

## 3. Materials and Methods

### 3.1. Reagents and Chemicals

Potassium hydroxide (KOH) flakes (≥90.0%), P.A. ethyl alcohol (≥99.9%), and P.A. deuterated chloroform (CDCl_3_) (≥99.8%) were purchased from Êxodo Científica (Sumaré, São Paulo, Brazil). The 5-fluorouracil (≥99.9%) and Gemcitabine (≥98.0%) chemotherapeutics, the P.A. dimethyl sulfoxide (DMSO) (≥99.5%), and the MTT salt (3-(4,5-dimethylthiazole-2-yl)-2,5-diphenyltetrazolium) were purchased from Sigma-Aldrich/Merck (Darmstadt, Germany). Dulbecco’s modified Eagle’s medium (DMEM), fetal bovine serum (FBS), and Penicillin (100 U/mL)/Streptomycin (10,000 units/mL of penicillin and 10,000 µg/mL of streptomycin) were purchased from Gibco^®^ (Grand Island, NY, USA). All reagents and chemicals were used as received with no further purification processing.

### 3.2. Plant Material and Obtention of Piperine Crystals

Dried seeds of black pepper (*Piper nigrum* L.), purchased from a local market of Abaetetuba city in the state of Pará, Brazil, were milled mechanically employing a porcelain crucible and pestle arrangement (A-100, Chiarotti, Mauá, São Paulo, Brazil). Afterward, we immersed 200.0 g of the crushed black pepper into 1.0 L of P.A. ethyl alcohol. The solution was stored at room temperature (~30 °C) for seven days until submitted to simple filtration to remove remnant milled pepper, resulting in the ethanolic extract of *Piper nigrum* (EEPN).

Considering that isolating natural molecules from extracts is generally a difficult endeavor in which undesired products (e.g., essential oils, isomers, and other metabolites) can be obtained concomitantly, we opted to employ three heating-independent recrystallization-based isolation methodologies to force piperine to crystallize and form solid-state samples (P1, P2, and P3). The obtention of P1 counts with the mixing, at room temperature, of a 100.0 mL EEPN aliquot with 10.0 mL of an aqueous KOH solution (4% *v*/*v*) and 40.0 mL of distilled water. The sample P2 followed a similar methodology, mixing a 100.0 mL EEPN aliquot with 10.0 mL of an ethanolic KOH solution (4% *v*/*v*) and 40.0 mL of distilled water. Finally, sample P3 originates from a green extraction method, free of KOH, in which a 100.0 mL EEPN aliquot is mixed directly with 50.0 mL of distilled water. All three mixtures were stored at room temperature for seven days forming yellow-colored solid precipitates after this period. The samples were filtered, and their solid phases (piperine crystals) were isolated for further characterization. Since there are no reports demonstrating that other components available in *Piper nigrum* L. extracts can produce yellow-colored needle-shaped crystals, we expect these substances to remain in the liquid phase, discarded after sample filtration. Six replicates were prepared to ensure the reproducibility of the proposed isolation methods.

### 3.3. Characterization Methods

#### 3.3.1. X-ray Diffraction (XRD)

The X-ray diffractograms were obtained with the samples in the solid-state in a BRUKER (Leipzig, Germany) diffractometer model D2 PHASER, equipped with a goniometer (θ/θ), radius: 141.1 nm, a copper anode ceramic X-ray tube (Cu-Kα1), and a 1D Lynxeye detector with a 5°, 2θ aperture and 192 channels. The characteristic emission line is located at 1.540598 Å/8.047 keV, with a maximum power of 300 W (30 kV × 10 mA).

#### 3.3.2. Optical Microscopy and Scanning Electron Microscopy (SEM) Coupled with Energy-Dispersive X-ray Spectroscopy (EDS)

Scanning electron micrographs were obtained for the solid-samples in a SEM microscope model TM 3000 Hitachi (Tokyo, Japan) coupled with a Energy-Dispersive X-ray Spectroscopy (EDS) TESCAN S8000 detector (Brno, Czech Republic), model VEGA TC. Optical microscopy analysis of the solid samples was performed employing a phase contrast optical microscope, Axio Observer 5 Zeiss (Oberkochen, Germany).

#### 3.3.3. Nuclear Magnetic Resonance (NMR)

The ^1^H and ^13^C NMR spectra were obtained using an instrument from Bruker (Leipzig, Germany), model Advance 400, which is equipped with a 5 mm cryogenic probe with 16 acquisitions each at a temperature of 25.7 °C. For the measurements, 30.0 mg of the P1, P2, and P3 samples were dissolved in deuterated chloroform (CDCl_3_). This solvent was used as an internal reference for the calibration of the equipment. The spectral widths for ^1^H and ^13^C were 15 ppm and 200 ppm, respectively.

#### 3.3.4. Infrared Spectroscopy

The infrared spectra of the solid-state P1, P2, and P3 samples were measured in a Fourier infrared spectrometer from Bruker (Leipzig, Germany), model Vertex 70v, which is equipped with a high efficiency interferometer (spectral resolution of ~1.0 cm^−1^) and a vacuum pump detector.

#### 3.3.5. UV–Vis Absorption and Photoluminescence (PL) Spectroscopies

Absorption spectra were acquired in a JASCO V-670 spectrophotometer (Easton, MD, USA) whereas steady-state photoluminescence (PL) spectra were measured in a Deltaflex TCSPC Lifetime Fluorometer from Horiba (Kyoto, Japan), equipped with a pulsed excitation source (λ_exc_ = 352 nm with 8.0 MHZ of repetition rate). All measurements were conducted in quartz cuvettes (1.0 cm path length) with the P1, P2, and P3 samples dissolved in P.A. dimethyl sulfoxide (DMSO).

### 3.4. In Vitro Cytotoxicity Activity

#### 3.4.1. Cell Culture

Gastric adenocarcinoma cell lines of the intestinal and diffuse Lauren’s types [112,113] were used, including AGP-01 (malignant ascites), AGP-01 *PIWIL1 KO* (*PIWIL1* gene inactivated) [82], ACP02 (primary cancer of the diffuse type), and ACP03 (primary cancer of the intestinal type), as well as non-neoplastic African Green Monkey Kidney (VERO) cell for comparison. Cells were grown in adherent monolayer cultures in Dulbecco’s modified Eagle’s medium (DMEM) supplemented with 10% fetal bovine serum, penicillin (100 U/mL), and streptomycin (100 mg/mL) and maintained at 37 °C in 5% carbon dioxide.

#### 3.4.2. Cytotoxicity Assay

The cytotoxic activity of the P1, P2, and P3 samples was evaluated for all lineages (AGP01, AGP01 *PIWIL1 KO*, ACP02, and ACP03) using the MTT colorimetric assay. The 5-fluorouracil (5-FU) and gemcitabine (GEM) chemotherapeutics were evaluated for AGP01 lineage. This assay is based on the conversion of the yellow MTT salt to formazan, a purple chromogenic product [114,115,116] by metabolically viable cells. All solid-state samples (P1, P2, and P3) were dissolved in dimethyl sulfoxide for the MTT assay. Cells were seeded in 96-well plates at a density of 10^3^ cells/well for 24 h to allow adhesion in the plate. The treatment was accomplished in a dose–response curve, with seven concentrations ranging from 1.56 μg/mL to 100 μg/mL, with further incubation at 37 °C for 72 h. The dissolved piperine samples in DMSO were added to the DMEM culture medium for cell treatment. The negative control was taken as the untreated cells, and the experiments were performed in triplicate. After treatment, 100 μL of MTT solution, 5 mg/mL stock solution, diluted 1:10 *v*/*v* in DMEM medium, was added to each well of the plate and incubated at 37 °C for 3 h. Absorbance of each plate was measured using a microplate spectrophotometer at 570 nm (SYNERGY/HT microplate reader, BioTek, Winooski, VT, USA).

Considering the individual IC50 values of P2 piperine (selected after the results analyses), of 5-FU and GEM, different concentrations of the chemotherapeutics (0.313–10 μg/mL) were combined to a constant concentration (IC50) of P2 (12 μg/mL), and the cell viability was obtained through of the MTT assay as described above.

#### 3.4.3. Data Analysis

A sigmoidal dose–response equation (non-linear regression) was used to determine the half maximal inhibitory concentration (IC50) and their respective confidence intervals (95% CI). Cell viability was obtained from the percentages relative to the negative control, using Equation (1), where *Abs_exp_* and *Abs_ctr_* account for the absorbance tracked at 570 nm for the experimental and control samples, respectively. To verify differences between the experimental groups, the ANOVA test (two way), followed by Bonferroni’s posttest, was performed, with significance levels *p* > 0.005 (**) and *p* > 0.0001 (***).

(1) $$Cell\;viability\;\left(\%\right)=\left(\frac{Abs_{exp}}{Abs_{ctr}}\right)*100$$

## 4. Conclusions

In summary, in the present study, three heating-independent extraction methods proved to be low-cost alternatives and reliable for the easy extraction of piperine crystals from black pepper seeds. These processes introduce new approaches to obtaining piperine. The samples originating from these processes (P1, P2, and P3) showed cytotoxic effects on gastric cancer cells, with P2 exhibiting the best cytotoxicity and selectivity. Furthermore, piperine was more active in cells with an aggressive phenotype, and possibly its mechanism of action involves cell migration and invasion pathways. This finding demonstrates for the first time the relationship between piperine cytotoxic activity and the phenotype of the gastric cancer lineages. Additionally, the results showed that besides acting in isolation on cancer models, when tested in combination, piperine provides considerable improvement in the activity of commercial chemotherapeutics such as 5-Fluorouracil and Gemcitabine, which is a very promising finding with possible outcomes in future research and clinical endeavors. This action leads to a decrease in the effective concentration of chemotherapeutic drugs on cells, which may mean more effective therapeutic perspectives and a reduction in toxicity. This set of results demonstrates the potential use of piperine in both isolated and combined forms in gastric cancer models, supporting the promising applicability of natural products in generating new therapies.

## Data Availability

Not applicable.
